# A Probable New Human Trypanosome

**Published:** 1911-02-25

**Authors:** 


### Tropical Medicine.

### A PROBABLE NEW HUMAN TRYPANOSOME.

Stephens and Fantham, in the "Annals of Tropical Medicine and Parasitology" for December 1910, describe what they believe to be a new human trypanosome. The parasite in question was obtained from a case who was infected in North-eastern Rhodesia, and who had never, as far as can be judged, been in an area where *Glossina palpalis* exists. Sub-inoculations into rats, monkeys, and other animals were made, and then it was found that amongst the stout or stumpy forms some had the nucleus at the posterior (non-flagellar) end. Such a phenomenon, the authors state, has never been found in animals infected with *G. palpalis*, either by themselves or by other investigators, and so they conclude that it is probable that they are dealing with a new species of trypanosome. Corroborative evidence of this is to be found in the fact that Dr. Yorke found that this Rhodesian strain was distinctly more virulent to rats, guinea pigs, and other laboratory animals than *T. gambiense*, and it also was strongly resistent to atoxyl. Professor Ross and Dr. Thomson, studying the case from which this trypanosome came, clinically found that a regular periodical increase in the numbers of the parasites took place in the peripheral blood of the patient from day to day.

